# Knowledge, attitudes, and practices on paediatric tuberculosis in rural Tanzania: insights for enhanced detection and care

**DOI:** 10.7189/jogh.16.04291

**Published:** 2026-10-23

**Authors:** Elisabetta Venturini, Giada Rinninella, Ludovica Facchini, Roberta Pellegrino, Andrea Miftode, Agnese Tamborino, Leila Bianchi, Francesca Corsi, Pendo Paschal Masanja, Stephen Matthew Kibusi, Alpha N Kwilasa, Hamoud Ndengue, Peres Lukango, Daniela Cuomo, Luca Scali, Maria José Caldés Pinilla, Luisa Galli

**Affiliations:** 1 Paediatric Infectious Diseases Unit, Meyer Children’s Hospital IRCCS, Florence, Italy; 2 Paediatric Infectious Diseases Unit, Luigi Sacco Hospital, Milan, Italy; 3 Meyer Children’s Hospital IRCCS, Florence, Italy; 4 Department of Paediatrics, AORN San Giuseppe Moscati, Avellino, Italy; 5 Experimental and Clinical Medicine Department, University of Florence, Italy; 6 Centro Mondialità Sviluppo Reciproco, Livorno, Italy; 7 School of Nursing and Public Health, University of Dodoma, Dodoma, Tanzania; 8 Regional TB and Leprosy Programme-Dodoma, United Republic of Tanzania Ministry of Health, Dodoma, Tanzania; 9 Global Health Centre, Tuscany Region, Florence, Italy; 10 Department of Health Sciences, University of Florence, Italy

**Keywords:** tuberculosis, children, knowledge-attitudes-practices (KAP), health workers, case detection, rural Tanzania

## Abstract

**Background:**

Paediatric tuberculosis (TB) remains underdiagnosed worldwide, with nearly 50% of cases missed. This issue is especially prominent in high-burden settings, where data on healthcare capacity for paediatric TB detection are limited. We assessed the knowledge, attitudes, and practices (KAP) regarding paediatric TB among healthcare workers (HCWs), community health workers (CHWs), and families in rural Tanzania.

**Methods:**

We conducted a cross-sectional KAP survey in Kondoa District (Dodoma Region) from November 2023 to October 2024. We measured knowledge and attitudes using validated questionnaires and assessed practices through reported TB-related activities.

**Results:**

Our sample included 87 HCWs, 86 CHWs, and 78 caregivers. Median knowledge scores (maximum = 18) were 11.2 (interquartile range (IQR) = 9.2–12.9) for HCWs and 8.9 (IQR = 7.9–9.8) for CHWs. Clinicians outperformed paramedical staff (*P* < 0.01), with those who had <5 years’ experience scoring better. Attitude scores were high at 3.25/4 for HCWs and 3.1/4 for CHWs, with a moderate knowledge-attitude correlation among HCWs (*ρ* = 0.344, *P* = 0.001). Practice gaps were substantial: 17.2% of HCWs never diagnosed paediatric TB, only 24% referred for advanced diagnostics, and 38% of CHWs reported no cases. Although 92% of caregivers were aware of TB, only 1.8% of children had documented diagnoses.

**Conclusions:**

Despite adequate knowledge, implementation gaps persist, suggesting systemic barriers. Strengthening diagnostics, referrals, training and stronger integration of CHWs into paediatric TB care pathways is essential.

According to the World Health Organization’s 2024 Global Tuberculosis Report, approximately one-quarter of the world’s population is infected with *Mycobacterium tuberculosis* [1]. Children aged 0–14 years account for approximately 12% of the global tuberculosis (TB) burden, with an estimated 1.3 million paediatric cases occurring annually [1]. Despite being preventable and treatable, paediatric TB remains largely underdiagnosed. In 2023, nearly 700,000 paediatric cases and approximately 200,000 TB-related deaths were reported globally, mostly among children under five years [1,2]. The case detection gap seems to be increasing with decreasing age, reaching up to 72% among children under five [2,3]. Diagnosis in children is challenging due to non-specific symptoms, higher rates of extrapulmonary and paucibacillary disease, and difficulties in obtaining sputum samples, often requiring invasive procedures such as gastric aspiration [4,5]. Primary health centres frequently lack adequate diagnostic tools, and clinicians may hesitate to initiate treatment without bacteriological confirmation [3,6,7]. Social stigma, limited awareness, and financial barriers further delay care-seeking and treatment adherence [8].

Healthcare workers (HCWs) and community health workers (CHWs) play a central role in improving early detection, treatment initiation, and contact tracing [9], with adequate knowledge and training among these cadres being associated with improved TB control outcomes [7,9,10]. Despite this, knowledge, attitude, and practice (KAP) surveys conducted in comparable sub-Saharan African settings have consistently documented important gaps: in Botswana, physicians demonstrated significantly better knowledge than other cadres, yet practice-level deficits persisted across all groups [11], while in Mozambique, HCWs showed limited awareness of paediatric-specific diagnostic approaches [12]; and a systematic review of KAP studies in low and middle-income countries found that positive attitudes did not reliably translate into appropriate practices, identifying structural barriers as the primary determinants of implementation gaps [10]. Community health workers have also been identified as an underutilised resource in TB care, with evidence that formal training and integration into referral pathways can improve case detection in high-burden settings [13]. Nevertheless, assessments of paediatric TB-related KAP simultaneously among HCWs, CHWs, and caregivers remains limited. Tanzania remains among the 30 high TB burden countries, and although the incidence of TB within the country declined from 306 per 100,000 population in 2015 to 183 per 100,000 population in 2023, paediatric TB remains a concern, with children accounting for 15.8% of notified TB cases in 2023, 98% of which were clinically diagnosed rather than bacteriologically confirmed, underscoring diagnostic challenges [14].

To address these gaps, we aimed to assess KAPs regarding paediatric TB among HCWs and CHWs in Kondoa District, Dodoma Region, Tanzania, and to evaluate awareness of paediatric TB among caregivers.

## METHODS

We conducted a KAP survey in the Kondoa District, Dodoma Region, Tanzania, from November 2023 to October 2024, within the ‘Community-Based TB and HIV Integration Project (CBTHI Project)’ led by *Centro Mondialità Sviluppo Reciproco*. The project, which had been registered in OpenAid [15], aimed to improve the accessibility and quality of care for TB and HIV in the region, particularly for children and adolescents.

According to the 2022 National Census, Kondoa District Council has a population of 244,854, mainly in remote rural villages; only 64.4% live within 5 km of a health facility, and about 40% lack reliable healthcare services. The Kondoa District has 39 health facilities (8 health centres and 31 dispensaries), of which 26 were involved in the CBTHI Project, covering approximately 115,050 people. At the time of the research, one of the district hospitals was under construction.

We enrolled HCWs and CHWs involved in TB care, and family members; all individuals working in the 26 CBTHI project facilities were invited to participate voluntarily, with no financial incentive or compensation being offered. To minimise response bias, we did not inform participants about the topic of the questionnaire prior to enrolment. The HCWs then completed a self-administered questionnaire, while CHW and family members were interviewed using structured questionnaires, with support from a local cultural mediator.

In the absence of prior KAP data from this specific district and population, and given the exploratory nature of the study and the logistical constraints of operating across 26 rural facilities, we could not define a reliable effect size estimate *a priori* on which to base a power calculation. The final sample should, therefore, be considered a census of available, consenting participants within the project facilities, rather than as adequately statistically powered, and subgroup comparisons should be interpreted with caution.

### Questionnaires

We used three questionnaires, each tailored to the target study population (Files S1, S2, and S3 in the **Online Supplementary Document**). The HCWs completed the validated KAP questionnaire developed by Joshi *et al.* [9], comprising 74 items covering sociodemographic characteristics, KAPs, satisfaction with local health services, community perceptions of TB, and two clinical vignettes. The CHWs completed an adapted version of the same questionnaire, for which we removed or simplified items requiring clinical decision-making or technical procedures, including gastric aspiration, antibiotic prescribing, treatment management, and clinical vignettes to reflect their non-clinical role. The adapted questionnaire retained the core KAP domains to allow comparison with HCW responses. It was reviewed by the study team and pilot-tested among a small group of CHWs to assess clarity, comprehension, and contextual relevance, after which we introduced minor wording revisions. We did not perform formal psychometric validation of the adapted instrument. Lastly, we developed a separate questionnaire for family members to assess awareness of paediatric TB, including knowledge of symptoms, transmission, and risk factors, perceptions of stigma, and awareness of free TB and HIV services. Caregivers of children with a previous TB diagnosis were additionally asked about their experience with diagnosis, treatment, adherence, adverse events, and HIV testing.

Knowledge assessment covered four domains: epidemiology, diagnosis, treatment, and prevention. Following the methodology described by Joshi *et al*. [9], we scored each item from 0 to 1, with multiple-response questions weighted proportionally according to the number of correct answers. We calculated domain-specific and overall knowledge scores for HCWs and CHWs.

We assessed attitudes towards TB risk, screening, diagnosis, and treatment using a four-point Likert scale and grouped into emotional, cognitive, and behavioural domains. An overall attitude score was calculated as the median of all attitude items.

Practices included screening, diagnosis, treatment, prevention and health education activities, contact tracing, and TB preventive therapy use, with responses recorded as frequency categories (‘always’, ‘often’, ‘sometimes’, or ‘never’) and summarised as percentages. We separately analysed family members’ responses descriptively and reported them as frequencies and percentages.

### Statistical analysis

We described categorical variables using frequencies and percentages, and analysed differences between groups analysed with chi-squared tests or Fisher’s exact tests, as appropriate. We reported continuous variables as medians and interquartile ranges (IQRs), and assessed differences between groups using the non-parametric Mann–Whitney U test. We used Spearman’s rank correlation analysis to examine the association between knowledge and attitude scores, with statistical significance set at *P* < 0.05.

For exploratory logistic regression analyses, we dichotomised knowledge scores using a threshold of 60% of the maximum attainable score. This cut-off was based on the modified Bloom’s criteria, which are widely used in KAP surveys to distinguish adequate from poor knowledge in the absence of validated questionnaire-specific thresholds [16-18].

Data were analysed using IBM SPSS Statistics, version 30.0.0.0 (IBM Corp., Armonk, New York, USA).

## RESULTS

A total of 173 participants completed the survey, with 50.3% being HCWs and 49.7% being CHWs (**Table 1**). Among 87 HCWs, 42.5% were clinicians and 57.5% paramedical staff; most worked in primary health centres (92%) as permanent staff (90%). All CHWs were volunteer workers in primary health centres; 44 (51%) had worked there for over 5 years and 35% for more than 10 years. The HCWs were younger and less experienced than CHWs (*P* < 0.001); 77% had <5 years’ experience, and all had received paediatric TB training within the previous two years. Nearly all (99%) of the 86 CHWs were community volunteers (61.6% female, median age of 43 years); they mainly completed primary education and were employed in agriculture. About 51% had >5 years’ experience and 99% had attended TB training in the past two years. Seventy-eight caregivers (63% female, median age of 39 years) were interviewed; most were parents, lived in rural areas, and worked in agriculture, with only 10% having completed secondary education.

**Table 1:** Sociodemographic characteristics of the study population, stratified by groups

	Total (n = 173)	CHWs (n = 86)	HCWs (n = 87)	*P*-value
**Age in years, Mdn (IQR)**	33 (29-45)	43 (32-52)	30 (28-33)	<0.001
**Gender**				0.22
Male	75 (43.4)	33 (40)	42 (48.3)	
Female	98 (56.6)	53 (61.6)	45 (51.7)	
**Contractual situation**				<0.001
Volunteer	89 (51.5)	85 (99)	4 (4.6)	
Temporary/permanent staff	84 (48.5)	1 (1)	83 (95.4)	
**Type of health facility**				NA
Primary health centre	166 (96)	86 (100)	80 (92)	
District hospital	7 (4)	0 (0)	7 (8)	
**Working experience**				<0.001
<5 years	109 (63)	42 (49)	67 (77)	
>5 years	64 (37)	44 (51)	20 (23)	

CHW – community health worker, HCW – healthcare worker, IQR – interquartile range, Mdn – median, NA – not applicable

*Presented as n (%) unless specified otherwise.

Seventy-eight caregivers participated in the survey (Table S2 in the **Online Supplementary Document**). The median age was 39 years (IQR = 31.0–51.8), and most respondents were mothers (49%) or fathers (31%). Educational attainment was generally low, with 76% reporting primary education as their highest level of schooling and 9% having received no formal education. Agriculture was the predominant occupation (81%). Households were typically large, with a median of five cohabitants (IQR = 4–7) and three children per household (IQR = 2–5). Nearly all participants resided in rural villages (99%).

### HCWs’ KAP questionnaire

The median knowledge score (maximum of 18) was 11.21 (IQR = 9.2–12.9), with the highest scores observed in the domains of epidemiology and treatment (**Table 2**). The HCWs with <5 years of experience scored about one point higher than those with >5 years (11.5 *vs*. 10.3; *P* = 0.14). Clinicians had significantly higher scores than other roles (*P* < 0.001). No differences emerged by gender, age, or attitude. Multivariate analysis showed no significant advantage for >10 years’ experience (**Table 3**). However, clinical officers and those trained in paediatric TB in the past two years scored significantly higher (*P* = 0.024; *P* = 0.036). The median attitude score was 3.2, with cognitive and behavioural domains >3. The HCWs with <5 years’ experience had slightly higher scores than those with >5 years (3.0 *vs*. 2.8; *P* = 0.06).

**Table 2:** Knowledge and attitude scores, stratified by study groups*

	Knowledge					Attitude			
	**Total score†**	**Epidemiology‡**	**Diagnosis‡**	**Treatment§**	**Prevention§**	**Total score§**	**Cognitive§**	**Emotional§**	**Behavioural§**
**CHWs (n = 86)**	8.87 (7.87–9.84)	3.49 (2.95–3.99)	1.84 (1.55–2.60)	2.00 (1.00–2.00)	1.5 (0.50–2.00)	3.1 (2.9–3.3)	3.4 (3.1–3.6)	3.0 (2.5–3.8)	3.3 (3.0–3.5)
Male	8.53 (7.16–9.57)	3.32 (2.99–3.98)	1.82 (1.57–2.61)	2.00 (1.37–2.00)	1.00 (0.50–2.00)	2.91 (2.75- 3.12)	3.50 (3.0–3.57)	2.75 (2.5–3.25)	3.25 (2.75–3.33)
*P-value*	0.08	0.71	1.00	0.50	0.20	0.05	0.43	0.46	0.03
Working experience >5 years	9.08 (7.99–10.29)	3.49 (2.86–3.99)	1.90 (1.46–2.59)	2.00 (2–2)	1.50 (0.50–2.37)	2.91 (2.78–3.1)	3.43 (3.14–3.57)	2.75 (2.45–3.0)	3.25 (2.9–3.5)
*P-value*	0.52	0.99	0.83	0.17	0.61	0.52	0.99	0.83	0.17
**HCWs (n = 87)**	11.21 (9.19–12.92)	3.66 (3.00–4.32)	2.27 (1.92–3.07)	3.00 (2.25–4)	2.50 (1.50–3.00)	2.9 (2.7–3.1)	3.3 (3.00–3.6)	2.8 (2.3–3.3)	3.00 (2.5–3.3)
Male	11.36 (9.42–14.01)	3.99 (3.16–4.32)	2.55 (2.02–3.16)	3.00 (2.25–4.00)	2.50 (1.50–3.00)	2.96 (2.77–3.2)	3.43 (3.07–3.57)	2.75 (2.25–3.25)	3.0 (2.5–3.25)
*P-value*	0.17	0.32	0.10	0.11	0.41	0.09	0.41	0.99	0.012
Working experience >5 years	10.89 (8.67–12.13)	3.24 (2.86–4.24)	2.16 (1.59–2.55)	3.00 (2.06–3.75)	2.00 (1.62–2.50)	2.8 (2.6–3.0)	3.3 (3.1–3.6)	2.5 (2.3–3.0)	2.8 (2.5–3.0)
*P-value*	0.14	0.08	0.17	0.69	0.20	0.06	0.87	0.06	0.24
Clinicians	12.07 (10.36–14.04)	4.15 (3.32–4.65)	2.47 (2.09–3.09)	3.00 (2.25–4.00)	2.50 (2.00–3.00)	2.96 (2.87–3.20)	3.43 (3.14–3.57)	3.0 (2.5–3.5)	3.0 (2.5–3.25)
*P-value*	<0.001	<0.001	0.04	0.08	<0.001	0.05	0.17	0.01	0.28

*Presented as median (interquartile range) unless specified otherwise.

†Maximum score is 18.

‡Maximum score is 5.

§Maximum score is 4.

**Table 3:** Univariate and multivariate predictors of higher knowledge scores (>60%)

	Univariate analysis		Multivariate analysis	
	**Unadjusted OR (95% CI)**	***P*-value**	**Adjusted OR (95% CI)**	***P*-value**
**HCWs**				
Gender	0.983 (0.411–2.348)	1.000		
Age >30 years	0.824 (0.346–1.954)	0.669	0.882 (0.312–2.469)	0.81
Clinical officer	2.328 (0.958–5.916)	0.078	3.527 (1.222–11.278)	0.024
Attitude (>2.9 out of 4)	2.047 (0.860–5.007)	0.128	1.873 (0.708–5.106)	0.209
Experience >10 years	0.331 (0.039–1.914)	0.218	0.113 (0.005–1.026)	0.08
Training on TB in the last two years	2.071 (0.687–7.218)	0.283	4.83 (1.241–25.462)	0.036
Previous research study	1.462 (0.458–5.261)	0.573		0.288
**CHWs**				
Gender	1.722 (0.659–4.822)	0.341	1.106 (0.895–1.369)	0.354
Age >30 years	0.712 (0.229–2.378)	0.563	0.872 (0.666–1.142)	0.322
Attitude (>2.9 out of 4)	0.888 (0.348–2.236)	0.820	0.925 (0.754–1.135)	0.458
Experience >10 years	1.149 (0.431–2.978)	0.810	1.089 (0.868–1.367)	0.461
Training on TB in the last two years	0.797 (0.261–2.634)	0.772	0.994 (0.770–1.283)	0.962
Someone in the family treated for TB	5.011 (1.789–14.801)	0.002	1.487 (1.193–1.853)	<0.001
Previous research study	0.453 (0.056–2.347)	0.441	1.215 (0.844–1.751)	0.298

CHW – community health worker, CI – confidence interval, HCW – healthcare worker, OR – odds ratio, TB – tuberculosis

We observed significant positive correlations among HCWs between total knowledge and all attitude domains (*ρ* = 0.344; *P* = 0.001). Treatment knowledge showed the most consistent associations, correlating significantly with all four attitude domains. Prevention knowledge was significantly correlated only with the emotional (*ρ* = 0.260; *P* = 0.015) and behavioural (*ρ* = 0.264; *P* = 0.014) domains. Overall, 14 out of 20 tested pairs (70%) were statistically significant (Table S1 in the **Online Supplementary Document**).

Clinical practices varied across the sample (**Figure 1**). Diagnosis of presumptive paediatric TB ranged from ‘never’ (17.2%) to ‘few times/week’ (24.1%). Treatment initiation occurred ‘few times/week’ (35.6%), ‘few times/month’ (31%), or ‘few times/year’ (14.9%). Most HCWs reported always providing TB education (72%), investigating child contacts (73%), tracing adult contacts (68%), and prescribing preventive therapy (62%). For sample collection, 56% collected sputum on the spot, 18% asked mothers to return with samples, and 24% referred elsewhere. Additionally, 43% performed gastric aspirates and 34% collected stool samples when sputum was unavailable.

**Figure 1:**
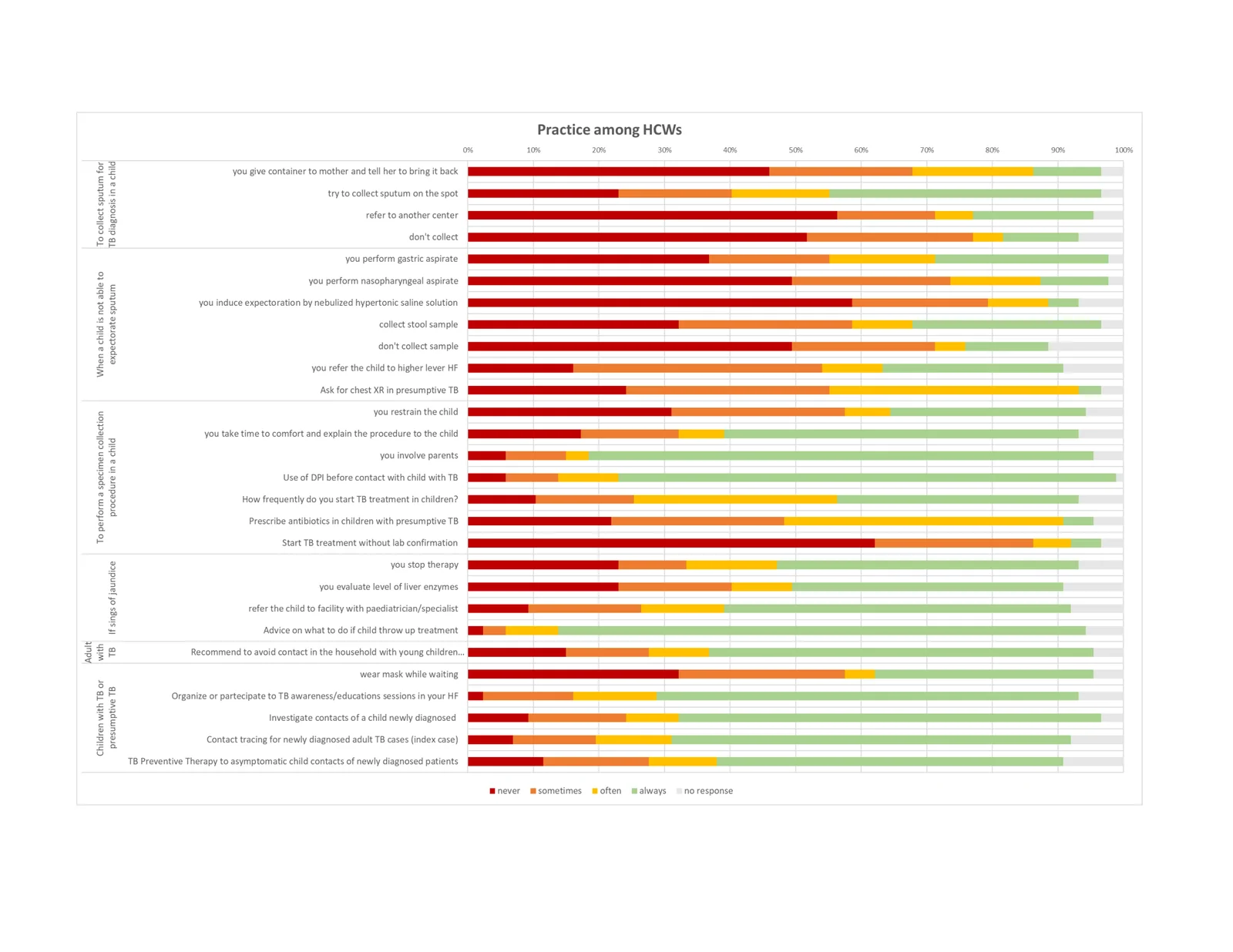
Practice among HCWs. HCW – healthcare worker, HF – hospital facility, TB – tuberculosis, XR – X-ray.

### CHWs’ modified KAP questionnaire

The median knowledge score (maximum of 18) among CHWs was 8.9 (IQR = 7.9–9.8), with highest results observed in the domains of epidemiology (3.5, IQR = 2.9–4.0) and treatment (2.0, IQR = 1.0–2.0), for which the maximum scores were 5 and 4, respectively (**Table 2**). Maximum scores were similar between women (9.2) and men (8.5). There were no differences (*P* = 0.125) in median scores between CHWs with ≤5 years of experience (11.56, IQR = 9.4–13.2) and those with >5 years (9.08, IQR = 7.99–10.29). This was subsequently confirmed in the multivariate analysis (**Table 3**). The CHWs with a family history of TB showed better understanding. The median attitude score was 3.1 (IQR = 2.9–3.3), highest in cognitive and behavioural domains. Those employed ≤5 years scored slightly higher (3.1 *vs*. 2.9; *P* = 0.04). We found no statistically significant correlations between any knowledge score or sub-domain and any attitude domain (*ρ* = −0.051; *P* = 0.641), with the correlation coefficients being uniformly close to zero (Table S1 in the **Online Supplementary Document**).

Clinical practices (**Figure 2**) showed that 38% of the CHWs had never encountered paediatric TB, while 8% had done so rarely, 35% very rarely, 13% monthly, and 6% weekly. Over half (55.6%) did not collect sputum in children and 47.6% referred them elsewhere. Most (89.5%) had never seen gastric aspirate performed, while 73% always collected stool if sputum was unavailable. About half consistently used PPE (58% masks), citing supply shortages. About 70% advised avoiding household contact, and 60% always conducted contact tracing for newly diagnosed children.

**Figure 2:**
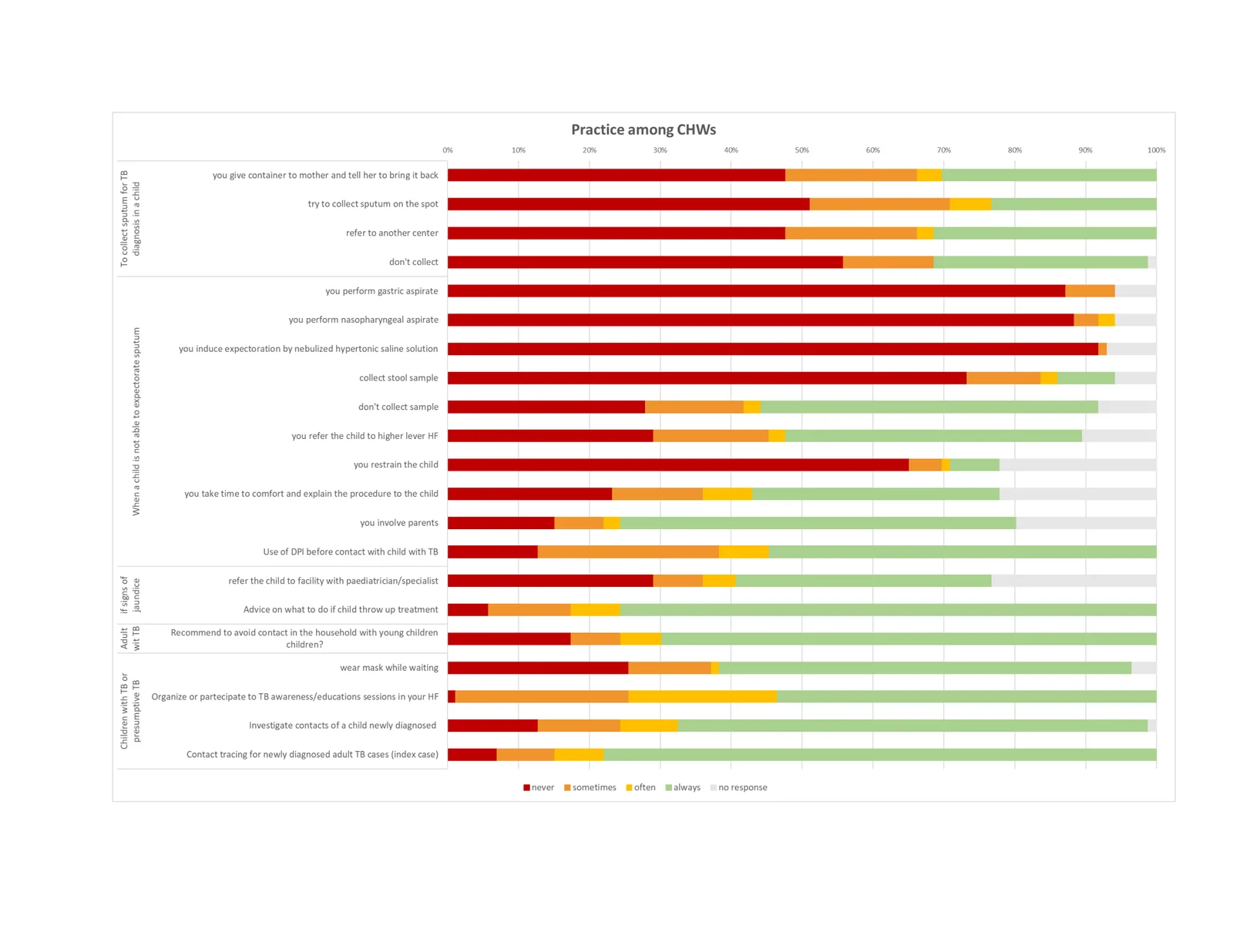
Practice among CHWs. CHW – community health worker, TB – tuberculosis, XR – X-ray.

### Families

Most respondents (n = 72, 92%) knew about TB, and 55% (n = 40) reported a TB case in their family or village. Over half were aware that HIV increases TB risk, 62% knew children are at higher risk, and about 70% recognised TB as contagious and treatment as free.

Among 281 children, 5 TB cases (1.8%) were reported; four had pulmonary TB and one had extrapulmonary TB. None were HIV-positive. Diagnosis was by sputum smear in 4 cases, with treatment lasting 6–12 months. HIV and TB screening was offered to three of the diagnosed children. Information regarding diagnosis, HIV testing, treatment duration, and treatment outcomes was based on caregiver report and was not independently verified through medical record review.

## DISCUSSION

This KAP survey conducted in the Kondoa District, Tanzania, provides insights into the KAPs regarding paediatric TB among HCWs, CHWs, and families in a high-burden, resource-limited setting. Our findings highlight a critical mismatch between knowledge and practice that must be addressed to improve paediatric TB detection and management in such contexts.

Knowledge scores among HCWs were moderate and comparable to international standard, including those reported by Joshi *et al*. [9], where Tanzanian participants performed similarly to Ugandan HCWs. This suggests that national training initiatives on paediatric TB may have contributed to the observed knowledge levels. Although the cross-sectional design does not allow their effectiveness to be assessed, the potential contribution of these initiatives is consistent with World Health Organization recommendations to strengthen healthcare worker capacity [7]. Clinicians showed significantly higher knowledge than paramedical staff, consistent with findings from Botswana, where physicians demonstrated significantly better knowledge than other healthcare categories [13]. While this pattern may reflect differences in formal educational preparation, alternative explanations, including cohort effects, differences in pre-service training curricula, and selection factors among professional groups should also be considered. Similarly, the observation that less experienced participants achieved higher knowledge scores should be interpreted cautiously and does not necessarily indicate that recent graduates are inherently better prepared. This pattern suggests that formal educational programmes may be more impactful than clinical experience, especially where paediatric TB is rarely encountered. Notably, the knowledge scores among CHWs (median 8.87/18) were remarkably close to those of HCWs, although the absolute scores remained modest.

According to the modified Bloom’s criteria adopted in this study, HCWs achieved knowledge levels classified as adequate, while CHWs’ scores remained below the predefined threshold. Given the diagnostic complexity of paediatric TB, these findings suggest that important knowledge gaps persist in both groups. The relatively similar scores between HCWs and CHWs may reflect the contribution of community-based training programmes and support their role as trained frontline providers and partners in TB control [11]. Practice findings reflect global challenges in paediatric TB diagnosis. Notably, 17.2% of HCWs reported never diagnosing presumptive TB, highlighting detection gaps linked to atypical presentations and sampling difficulties [4]. This gap has been highlighted by Rahman *et al*. [3], who identified innovative approaches across 18 countries to address similar challenges.

Sample collection practices varied: only 24% referred to specialised centres, while 56% collected sputum on-site. Limited access to tools like GeneXpert at peripheral facilities remains a major barrier. This finding aligns with observations from Mozambique, where diagnostic capacity limitations at primary healthcare level were identified as major obstacles [12]. The mention of alternative methods, such as gastric aspiration and stool sampling, suggest that these approaches are recognised, but raise concerns about feasibility and quality in peripheral settings. Recent advances in stool-based TB diagnostics offer promising solutions for resource-limited settings, though implementation requires substantial infrastructure development [5]. The reported involvement of CHWs in stool sampling requires careful interpretation. In practice, CHWs are more likely to advise caregivers on sample collection, support referral processes, and facilitate access to diagnostic services rather than independently perform specimen collection. Therefore, these findings should be interpreted as reflecting participation in the diagnostic pathway rather than direct technical responsibility for paediatric TB sample collection.

Furthermore, CHWs demonstrated strong involvement in contact tracing and activities related to case identification and referral, yet 38% had never encountered paediatric TB. This paradox reflects the broader challenge of low case detection rates in community settings, consistent with global patterns which show that paediatric TB remains largely undiagnosed [1,2]. The correlation between personal TB experience and better knowledge scores among CHWs suggests that experiential learning remains valuable, supporting findings from South Africa where home-based carers with direct TB exposure demonstrated superior knowledge [19]. However, the reliance on personal experience as a primary learning mechanism highlights the need for more systematic training approaches.

Attitudes were generally positive, particularly among recently trained staff. The moderate positive correlation between knowledge and attitudes among HCWs supports the premise that higher knowledge levels were associated with more positive attitudes toward TB. However, no causal relationship can be inferred from these cross-sectional data. The high level of TB awareness among families (92% knew about TB disease) represents a significant asset for community-based interventions. However, the low reported paediatric TB prevalence (1.8% among 281 children) may indicate continued underdiagnosis and underreporting rather than true low incidence, given Tanzania's status as a high-burden country with an estimated 20 TB cases per 100,000 children [14]. By including caregivers alongside HCWs and CHWs, this study provides a broader perspective on paediatric TB awareness and related challenges across different levels of the care pathway, an aspect that has received limited attention in previous KAP studies.

Several limitations must be acknowledged when interpreting these findings. The self-administered questionnaire format for HCWs may have introduced response bias. The involvement of multiple interviewers and translators could have affected consistency in the application of the questionnaires, especially for CHW and family interviews conducted in local languages. The timing of data collection following recent training programmes for some participants may have inflated knowledge scores, potentially overestimating baseline capacity. The reliance on self-reported practices rather than direct observation represents a common limitation in KAP studies, as noted in similar assessments from Nepal and Mozambique [12,20]. Actual clinical practices may differ significantly from reported behaviours, particularly in resource-constrained settings in which ideal practices may not be feasible. The considerable length of the questionnaire may have led to respondent fatigue, potentially compromising the quality of later responses. The reported difficulty in utilising graded response scales and a tendency for respondents to provide definitive ‘yes/no’ answers, rather than admitting uncertainty could further skew the attitude and practice data.

We did not design our study around a predefined sample size calculation, which may have made it underpowered to detect modest associations, particularly in subgroup analyses. Although all eligible and consenting participants within the participating facilities were enrolled, the relatively small sample size limits the precision of some estimates and increases the risk of type II error. Consequently, non-significant findings should not be interpreted as evidence of the absence of an association. The exploratory nature of the analyses means that observed associations require confirmation in larger, adequately powered studies. Furthermore, given the cross-sectional design, associations observed between training, experience, and KAPs should not be interpreted as evidence of causality.

We dichotomised knowledge scores using a conventional 60% threshold based on modified Bloom’s criteria. Although widely used in KAP research, this cut-off has not been specifically validated for the study instrument and alternative approaches, including modelling knowledge as a continuous outcome, may yield different estimates.

Overall, despite HCWs meeting the predefined threshold for adequate knowledge, their absolute scores remained modest, while CHWs scored below this threshold, indicating persistent knowledge gaps across cadres. These gaps, together with the reported challenges in paediatric TB detection and sample collection, highlight the need to strengthen both healthcare worker capacity and diagnostic pathways. Consistent with the findings of Rahman *et al*. [3] and the broader KAP literature [10], key priorities include continuous, skills-based training, improved access to diagnostic tools at peripheral facilities, and clearer referral pathways. In Kondoa District, these priorities could be addressed by integrating paediatric TB modules into routine CHW training, strengthening diagnostic capacity at district-level facilities, and establishing clear, supervised referral protocols between community and facility levels, in line with the World Health ORganization Roadmap for ending TB in children [7].

## CONCLUSIONS

This study provides one of the few comprehensive assessments of paediatric TB-related KAP across multiple cadres (HCWs, CHWs, and families) in a rural Tanzanian district. The findings suggest that awareness of paediatric TB and generally positive attitudes are present across the care pathway, although important knowledge gaps remain, particularly among CHWs. Despite this foundation, we identified substantial practice gaps, and the results are consistent with the influence of structural barriers, including limited diagnostic capacity, insufficient skills-based training, and weaknesses in referral systems. These findings have direct implications for the National TB and Leprosy Programme, supporting the prioritisation of skills-based training, expansion of point-of-care diagnostic services, and stronger integration of CHWs into paediatric TB care pathways. Longitudinal evaluation of targeted training interventions and qualitative exploration of implementation barriers would be important next steps to inform scalable, context-adapted strategies for improving paediatric TB outcomes in sub-Saharan Africa.

## Additional material


Online Supplementary Document

## Data Availability

**Data availability:** The data supporting the findings of this study are available from the corresponding author upon reasonable request.
