# Supplementary material for: Knowledge, attitudes, and practices on paediatric tuberculosis in rural Tanzania: insights for enhanced detection and care
Source: J Glob Health. 2026 Oct 23;16:04291. doi: 10.7189/jogh.16.04291 (PMC13693297; doi:10.7189/jogh.16.04291)
Supplement: Supplementary file 1 [file jogh-16-04291-s001.pdf]

Supplement to: Venturini E, Rinninella G, Facchini L, Pellegrino R, Miftode A, Tamborino A, Bianchi L, Corsi F, Masanja PP, Kibusi SM, Kwilasa AN, Ndengue H, Lukango P, Cuomo D, Scali L, Pinilla MJC, Galli L. Knowledge, attitudes, and practices on paediatric tuberculosis in rural Tanzania: insights for enhanced detection and care. J Glob Health. 2026;16:04291.

Table S1. Spearman's correlations between knowledge domains and attitude domains (global and sub-domains) among healthcare workers (HCWs) and community health workers (CHWs).

KNOWLEDGE DOMAIN	HCW				CHW			
	Global attitude ρ (p)	Cognitive ρ (p)	Emotional ρ (p)	Behavioural ρ (p)	Global attitude ρ (p)	Cognitive ρ (p)	Emotional ρ (p)	Behavioural ρ (p)
**Total knowledge**	**0.344 (0.001)**	**0.238 (0.026)**	**0.326 (0.002)**	**0.450 (<0.001)**	-0.051 (0.641)	-0.117 (0.285)	-0.020 (0.855)	0.066 (0.548)
**Epidemiology**	**0.260 (0.015)**	0.161 (0.137)	0.274 (0.010)	**0.271 (0.011)**	0.148 (0.173)	0.083 (0.450)	0.136 (0.212)	0.165 (0.128)
**Diagnosis**	**0.243 (0.024)**	0.177 (0.101)	0.161 (0.138)	**0.426 (<0.001)**	-0.059 (0.590)	-0.050 (0.648)	-0.163 (0.133)	0.078 (0.474)
**Treatment**	**0.421 (<0.001)**	**0.287 (0.007)**	**0.325 (0.002)**	**0.379 (<0.001)**	0.035 (0.748)	-0.009 (0.936)	0.087 (0.424)	-0.077 (0.480)
**Prevention**	0.095 (0.382)	0.037 (0.736)	**0.260 (0.015)**	**0.264 (0.014)**	-0.126 (0.247)	-0.133 (0.224)	-0.037 (0.736)	0.012 (0.913)

Values are Spearman's rank correlation coefficients (ρ) with corresponding p-values. Statistically significant associations (p<0.05) are shown in bold.

Table S2. General characteristics of the families enrolled in the study.

**POPULATION**	
**Gender**	
M	29 (37%)
F	49 (63%)
**Age**, years (median, IQR)	39 (31-51.75)
**RELATIONSHIP WITH THE CHILD**	
Mother	38 (49%)
Father	24 (31%)
Sister/brother	3 (4%)
Grandparents	13 (17%)
Household	72 (89.74%)
**EDUCATION**	
Primary	59 (76%)
Secondary	8 (10%)
No formal education	7 (9%)
**EMPLOYMENT**	
Agriculture	63 (81%)
Trade	9 (12%)
**TYPE of VILLAGE**	
Rural	77 (99%)
**COHABITANTS**	
Number of cohabitants (median, IQR)	5 (4-7)
Number of children in the household (median, IQR)	3 (2-5)
Age of children in the household (median, IQR)	8.75 (5-12.2)

TEST

" Strategy collaborative TB/HIV training for HCW" (30 minutes)

1. What is your gender? ☐ Male ☐ Female
2. What is your age? Please write your birth date and/or your age in years.
|___|___|___|___| / |___|___| AND/OR Age: |___|___| years

3. In what type of health facility do you currently work?
□ District Hospital
□ Primary Health Centre
4. What is your position within this health facility??
□ Community Health Worker/volunteer
□ Nursing assistant
□ Nurse
□ Midwife
□ Clinical Officer or Clinical Health Officer or Medical Assistant
□ General Practitioner
□ Paediatrician
□ Radiologist
□ Radiology Technician
□ Laboratory Technician or microscopist
□ Social assistant or counsellor
□ Other (specify) …………………………………………………………….
5. What is your contractual situation?
□ Permanent staff
□ Temporary staff on short-term contract or locum
□ Volunteer
□ Other (specify) ……………………………………………………
6. How long have you worked within this health facility?
□ Less than 1year
□ 1 to 5 years
□ 5 to 10 years
□ More than 10 years
7. In the last 2 years, did you receive any training on childhood TB? ☐ No ☐ Yes
8. Have you ever been sick with TB yourself? ☐ No ☐ Yes

9. Has someone you are in close contact with ever been treated for TB (for example: family members,spouse, etc.)? ☐ No ☐ Yes

10. Have you already worked or participated in a research study before? ☐ No ☐ Yes

Knowledge

11. What causes TB?
□ A bacteria
□ A virus
□ A fungus
12. How does TB spread?
□ Sexually
□ Through the air, when a person with TB coughs, speaks, or sings
□ By direct contact (shaking hand, kissing, sharing food, touching surfaces with *M. tuberculosis*)
□ By sharing needles
13. Among people who are in contact with someone with TB disease *(tick all that apply):*
□ All will develop TB disease quickly
□ Some can be infected by TB without developing the disease

□ All are protected from TB
□ Some will develop TB disease quickly
□ Some may develop TB disease years later
14. Which groups of children are at high risk of developing TB? (*tick all that apply*)
□ Children aged less than 1 year
□ Children living with HIV
□ Children aged 5-10 years
□ Children with severe acute malnutrition
15. What are common TB symptoms in children? (*tick all that apply*)
□ Cough for more than 2 weeks
□ Diarrhea
□ Persistent fever
□ Loss of appetite
□ Headache
□ Night sweats
□ Cutaneous rash
□ Weight loss
16. How is TB confirmed in adults? (*tick all that apply*)
□ Blood culture
□ Skin test
□ Blood rapid test
□ Chest radiography (CXR)
□ Sputum microscopy or Xpert MTB/RIF
□ Culture
17. How is TB diagnosed in children? *(tick all that apply)*
□ Blood culture
□ Chest X-ray
□ Skin test
□ Blood rapid test
□ Clinical diagnosis
□ Xpert MTB/RIF or Xpert Ultra on bacteriological sample
□ Culture
18. What kind of bacteriological samples are possible for TB diagnosis in children? *(tick all that apply)*
□ Expectorated sputum
□ Urine
□ Gastric aspirate
□ Nasopharyngeal aspirate
□ Cerebrospinal fluid
□ Stool samples
□ Induced sputum
□ Blood samples
19. What is the Xpert MTB/RIF test?
□ A test for CD4 lymphocytes count
□ A type of X-ray to diagnose TB
□ A molecular test for TB
□ A skin tuberculosis test
20. How many drugs are used in the initial (intensive) phase of TB treatment in children?
□ 1 or 2
□ 3 or 4
□ 5 or 6
□ 7 or 8
21. What drug(s) are parts of the initial (intensive) phase of tuberculosis treatment in children? *(tick all that apply)*
□ Isoniazid (INH)
□ Rifampicin (RIF)

□ Ciprofloxacin (CIP)
□ Levofloxacin (LEV)
□ Capreomycin (CAP)
□ Kanamycin (KAN)
□ Ethambutol (EMB)
□ Pyrazinamide (PZA)
□ Amikacin (AMK)
22. How long is the total duration of TB treatment in children?
□ 2 weeks
□ 2 months
□ 4 months
□ 6 months
□ 12 months
23. What could be the possible consequences of not completing TB treatment in children?
□ Development of resistant tuberculosis
□ Failure to fully cure the disease
□ Further transmission of the disease
□ All of the above
24. In children, TB is frequently associated with (*tick all that apply):*
□ Asthma
□ HIV
□ Malaria
□ Severe Acute Malnutrition
□ Diabetes
25. What is the name of the vaccine for TB?
□ ___________________________
□ I don't know
26. Does the vaccine protect fully against all forms of TB?
□ Yes
□ No
27. What is contact tracing? *(tick all that apply)*
□ Searching for people who were living in the same household with a patient diagnosed with TB (index case)
□ Searching for people who were in regular contact (school, work…) with a patient diagnosed with TB (index case)
□ Searching for people who were living in the neighbourhood a patient diagnosed with TB (index case)
□ Searching for anybody who has met at least once a patient diagnosed with TB (index case)
28. Who should receive TB preventive treatment among those following groups? *(tick all that apply)*
□ Children under 5 years old with a history of contact with TB
□ Children over 5 years old, adolescent and adults with a history of contact with TB
□ HIV-negative adults with a history of contact with TB
□ Children living with HIV without TB symptoms
□ Adults with HIV with TB symptoms

C. Attitudes

29. I am worried about being in contact with patients with TB
□ Strongly agree
□ Agree
□ Disagree
□ Strongly disagree
30. If I have TB symptoms or signs, I should be screened for TB
□ Strongly agree
□ Agree
□ Disagree

□ Strongly Disagree
31. lf I have TB symptoms or signs, l should wear a mask at the health facility
□ Strongly agree
□ Agree
□ Disagree
□ Strongly disagree
32. lf I have TB symptoms or signs, l should wear a mask/scarf at home
□ Strongly agree
□ Agree
□ Disagree
□ Strongly disagree
33. Any child attending outpatient clinics should be systematically screened for TB
□ Strongly agree
□ Agree
□ Disagree
□ Strongly disagree
34. I would not accept to examine a child with suspected TB
□ Strongly agree
□ Agree
□ Disagree
□ Strongly disagree
35. I believe TB diagnosis in children is more difficult than in adults
□ Strongly agree
□ Agree
□ Disagree
□ Strongly Disagree
36. I feel reluctant to collect induced sputum / throat aspirates (Nasopharyngeal Aspirates) among children
□ Strongly agree
□ Agree
□ Disagree
□ Strongly Disagree
37. I feel reluctant to collect and test stools for TB in children
□ Strongly agree
□ Agree
□ Disagree
□ Strongly Disagree
38. Invasive methods can be used in children to diagnose TB
□ Strongly agree
□ Agree
□ Disagree
□ Strongly Disagree
39. Gastric aspirate is an invasive method to use in children
□ Strongly agree
□ Agree
□ Disagree
□ Strongly Disagree
40. I would recommend to stop treatment if a child with TB is feeling better
□ Strongly agree
□ Agree
□ Disagree
□ Strongly disagree
41. Traditional medicine can be used in children treated for TB
□ Strongly agree
□ Agree

□ Disagree
□ Strongly Disagree
42. It is important to conduct contact investigation to identify children with TB or at risk of TB
□ Strongly agree
□ Agree
□ Disagree
□ Strongly disagree
43. Providing TB preventive therapy to children is important
□ Strongly agree
□ Agree
□ Disagree
□ Strongly disagree
Satisfaction with quality of care provided in the health facility
44. The majority of staff in my health facility have adequate training regarding childhood TB
□ Strongly agree
□ Agree
□ Disagree
□ Strongly Disagree
45. Diagnostic tools available in my health facility are adequate for the diagnosis of childhood TB
□ Strongly agree
□ Agree
□ Disagree
□ Strongly Disagree
46. Laboratory services in my health facility or that my health facility uses are adequate for the diagnosis of childhood TB
□ Strongly agree
□ Agree
□ Disagree
□ Strongly Disagree
47. In my health facility, laboratory test results are available on time to be able to decide to treat children for tuberculosis
□ Strongly agree
□ Agree
□ Disagree
□ Strongly Disagree
48. In my health facility, child-friendly fixed dose combination formulations are always available
□ Strongly agree
□ Agree
□ Disagree
□ Strongly disagree
49. In this health centre, it is complicated to refer children with presumptive tuberculosis to another health centre
□ Strongly agree
□ Agree
□ Disagree
□ Strongly Disagree
Community perceptions
50. People in my community believe that a child who has persistent cough should be brought to the clinic as soon as possible
□ Strongly agree
□ Agree
□ Disagree
□ Strongly Disagree
51. People in my community are worried about being in contact with children with TB or presumptive TB
□ Strongly agree

□ Agree
□ Disagree
□ Strongly Disagree
52. People in my community are aware of the availability of tuberculosis services in this health centre
□ Strongly agree
□ Agree
□ Disagree
□ Strongly Disagree

D. Practices

53. How often do you diagnose children with TB or presumptive TB in your health facility?
□ Few times a week
□ Few times a month
□ Few times a year
□ Never
54. To collect sputum for TB diagnosis in a child:
a) You give a container to the mother and you tell her to bring it back with sputum
□ Always
□ Often
□ Sometimes
□ Never
b) You try to collect sputum on the spot/immediately
□ Always
□ Often
□ Sometimes
□ Never
c) You refer to another center for sputum collection
□ Always
□ Often
□ Sometimes
□ Never
d) You don't collect sputum from children
□ Always
□ Often
□ Sometimes
□ Never
55. When a child has presumptive TB and is not able to expectorate sputum:
a) You perform gastric aspirate
□ Always
□ Often
□ Sometimes
□ Never
b) You perform nasopharyngeal aspirate
□ Always
□ Often
□ Sometimes
□ Never
c) You induce expectoration by nebulized hypertonic saline solution
□ Always
□ Often
□ Sometimes
□ Never
d) You collect stool sample
□ Always
□ Often

□ Sometimes
□ Never
e) You don't collect sputum
□ Always
□ Often
□ Sometimes
□ Never
f) You refer the child to higher level health facility
□ Always
□ Often
□ Sometimes
□ Never
56. Do you ask for chest X-Rays in children with presumptive TB ?
□ Never
□ Only if respiratory samples are negative
□ Case by case, according to children's condition
□ Systematically in all children
57. Do you use the following methods to perform a specimen collection procedure in a child?
a) Restrain the child during the procedure
□ Always
□ Often
□ Sometimes
□ Never
b) Take time to comfort and explain the procedure to the child
□ Always
□ Often
□ Sometimes
□ Never
c) Involve parents during the procedure
□ Always
□ Often
□ Sometimes
□ Never
58. Do you wear personal protective equipment before contact with children with TB or presumptive TB?
□ Always
□ Often
□ Sometimes
□ Never
59. How frequently do you initiate children on TB treatment?
□ A few times a week
□ A few times a month
□ A few times a year
□ Never
60. Do you prescribe a course of antibiotics in children with presumptive TB?
□ Never
□ Only if respiratory samples are negative
□ Case by case, according to children's condition
□ Systematically in all children
61. Do you start children on TB treatment without laboratory confirmation?
□ Always
□ Often
□ Sometimes
□ Never
62. What do you do if a child has yellowness of the eyes/skin after three weeks of TB treatment?
a) You stop treatment

□ Always
□ Often
□ Sometimes
□ Never
b) You evaluate level of liver enzymes if possible
□ Always
□ Often
□ Sometimes
□ Never
c) You refer the child to facility with paediatrician/specialist
□ Always
□ Often
□ Sometimes
□ Never
63. Do you give advice/education to parents/guardians on what to do when the child throws up the medicine?
□ Always
□ Often
□ Sometimes
□ Never
64. When an adult patient has TB, do you recommend to avoid contact in the household with young children?
□ Always
□ Often
□ Sometimes
□ Never
65. Do you recommend to children with TB or presumptive TB to wear a mask when waiting at the health facility?
□ Always
□ Often
□ Sometimes
□ Never
66. Do you organize or participate in TB awareness or education sessions in your health facility?
□ Always
□ Often
□ Sometimes
□ Never
67. Do you do investigation of contacts of a child newly diagnosed with TB?
□ Always
□ Often
□ Sometimes
□ Never
68. Do you do child contact tracing for newly diagnosed adult TB cases (index case)?
□ Always
□ Often
□ Sometimes
□ Never
69. Do you provide TB Preventive Therapy to asymptomatic child contacts of newly diagnosed patients?
□ Always
□ Often
□ Sometimes
□ Never
A child aged 3 years comes in with a persistent cough of 16 days duration, the cough is dry and the child is not short of breath. According to his mother, the child is less playful, looks very tired and has been feeling the same way for the past four weeks.

70. What likely diagnosis do you suspect?
□ HIV
□ Acute respiratory infection/pneumonia
□ Asthma
□ Tuberculosis
71. How would you confirm your likely diagnosis?
□ Ask for recent contact with TB patient
□ Clinical evaluation
□ HIV test
□ Xpert test
□ Malaria RTD
□ Tuberculin Skin Test
□ X-ray
A 6-year old child presents at the health facility after 1 week of antibiotics. He's feeling weak and he has been coughing for 4 weeks now. The mother explains that she came 10 days ago and that a test was done, it was negative. In your register you see that a sputum smear was done and it was negative.
72. Do you refer the child for chest-X-Ray?
□ Always
□ Often
□ Sometimes
□ Never
73. Do you send the child home with a new course of antibiotics?
□ Always
□ Often
□ Sometimes
□ Never
74. Do you try to do another TB test/refer for another TB test?
□ Always
□ Often
□ Sometimes
□ Never

TEST
" Strategy collaborative TB/HIV training for CHW" (30 minutes)

1. What is your gender? ☐ Male ☐ Female
2. What is your age? Please write your birth date and/or your age in years.
|___|___|___|___| / |___|___| AND/OR Age: |___|___| years

3. In what type of health facility do you currently work?
□ District Hospital
□ Primary Health Centre
4. What is your position within this health facility??
□ Community Health Worker/volunteer
□ Nursing assistant
□ Nurse
□ Midwife
□ Clinical Officer or Clinical Health Officer or Medical Assistant
□ General Practitioner
□ Paediatrician
□ Radiologist
□ Radiology Technician
□ Laboratory Technician or microscopist
□ Social assistant or counsellor
□ Other (specify) …………………………………………………………….
5. What is your contractual situation?
□ Permanent staff
□ Temporary staff on short-term contract or locum
□ Volunteer
□ Other (specify) ……………………………………………………
6. How long have you worked within this health facility?
□ Less than 1year
□ 1 to 5 years
□ 5 to 10 years
□ More than 10 years
7. In the last 2 years, did you receive any training on childhood TB? ☐ No ☐ Yes
8. Have you ever been sick with TB yourself? ☐ No ☐ Yes

9. Has someone you are in close contact with ever been treated for TB (for example: family members,spouse, etc.)? ☐ No ☐ Yes

10. Have you already worked or participated in a research study before? ☐ No ☐ Yes

Knowledge
11. What causes TB?
□ A bacteria
□ A virus
□ A fungus
12. How does TB spread?
□ Sexually
□ Through the air, when a person with TB coughs, speaks, or sings
□ By direct contact (shaking hand, kissing, sharing food, touching surfaces with *M. tuberculosis*)
□ By sharing needles
13. Among people who are in contact with someone with TB disease *(tick all that apply):*
□ All will develop TB disease quickly
□ Some can be infected by TB without developing the disease

□ All are protected from TB
□ Some will develop TB disease quickly
□ Some may develop TB disease years later
14. Which groups of children are at high risk of developing TB? (*tick all that apply*)
□ Children aged less than 1 year
□ Children living with HIV
□ Children aged 5-10 years
□ Children with severe acute malnutrition
15. What are common TB symptoms in children? (*tick all that apply*)
□ Cough for more than 2 weeks
□ Diarrhea
□ Persistent fever
□ Loss of appetite
□ Headache
□ Night sweats
□ Cutaneous rash
□ Weight loss
16. How is TB confirmed in adults? (*tick all that apply*)
□ Blood culture
□ Skin test
□ Blood rapid test
□ Chest radiography (CXR)
□ Sputum microscopy or Xpert MTB/RIF
□ Culture
17. How is TB diagnosed in children? *(tick all that apply)*
□ Blood culture
□ Chest X-ray
□ Skin test
□ Blood rapid test
□ Clinical diagnosis
□ Xpert MTB/RIF or Xpert Ultra on bacteriological sample
□ Culture
18. What kind of bacteriological samples are possible for TB diagnosis in children? *(tick all that apply)*
□ Expectorated sputum
□ Urine
□ Gastric aspirate
□ Nasopharyngeal aspirate
□ Cerebrospinal fluid
□ Stool samples
□ Induced sputum
□ Blood samples
19. What is the Xpert MTB/RIF test?
□ A test for CD4 lymphocytes count
□ A type of X-ray to diagnose TB
□ A molecular test for TB
□ A skin tuberculosis test
20. How many drugs are used in the initial (intensive) phase of TB treatment in children?
□ 1 or 2
□ 3 or 4
□ 5 or 6
□ 7 or 8
21. What drug(s) are parts of the initial (intensive) phase of tuberculosis treatment in children? *(tick all that apply)*
□ Isoniazid (INH)
□ Rifampicin (RIF)

□ Ethambutol (EMB)
□ Pyrazinamide (PZA)
22. How long is the total duration of TB treatment in children?
□ 2 weeks
□ 2 months
□ 4 months
□ 6 months
□ 12 months
23. What could be the possible consequences of not completing TB treatment in children?
□ Development of resistant tuberculosis
□ Failure to fully cure the disease
□ Further transmission of the disease
□ All of the above
24. In children, TB is frequently associated with (*tick all that apply):*
□ Asthma
□ HIV
□ Malaria
□ Severe Acute Malnutrition
□ Diabetes
25. What is the name of the vaccine for TB?
□ ____________________________
□ I don't know
26. Does the vaccine protect fully against all forms of TB?
□ Yes
□ No
27. What is contact tracing? *(tick all that apply)*
□ Searching for people who were living in the same household with a patient diagnosed with TB (index case)
□ Searching for people who were in regular contact (school, work…) with a patient diagnosed with TB (index case)
□ Searching for people who were living in the neighbourhood a patient diagnosed with TB (index case)
□ Searching for anybody who has met at least once a patient diagnosed with TB (index case)
28. Who should receive TB preventive treatment among those following groups? *(tick all that apply)*
□ Children under 5 years old with a history of contact with TB
□ Children over 5 years old, adolescent and adults with a history of contact with TB
□ HIV-negative adults with a history of contact with TB
□ Children living with HIV without TB symptoms
□ Adults with HIV with TB symptoms

C. Attitudes

29. I am worried about being in contact with patients with TB
□ Strongly agree
□ Agree
□ Disagree
□ Strongly disagree
30. If I have TB symptoms or signs, I should be screened for TB
□ Strongly agree
□ Agree
□ Disagree
□ Strongly Disagree
31. If I have TB symptoms or signs, I should wear a mask at the health facility
□ Strongly agree
□ Agree
□ Disagree

□ Strongly disagree
32. If I have TB symptoms or signs, I should wear a mask/scarf at home
□ Strongly agree
□ Agree
□ Disagree
□ Strongly disagree
33. Any child attending outpatient clinics should be systematically screened for TB
□ Strongly agree
□ Agree
□ Disagree
□ Strongly disagree
34. I would not accept to examine a child with suspected TB
□ Strongly agree
□ Agree
□ Disagree
□ Strongly disagree
35. I believe TB diagnosis in children is more difficult than in adults
□ Strongly agree
□ Agree
□ Disagree
□ Strongly Disagree
36. I feel reluctant to collect induced sputum / throat aspirates (Nasopharyngeal Aspirates) among children
□ Strongly agree
□ Agree
□ Disagree
□ Strongly Disagree
37. I feel reluctant to collect and test stools for TB in children
□ Strongly agree
□ Agree
□ Disagree
□ Strongly Disagree
38. Invasive methods can be used in children to diagnose TB
□ Strongly agree
□ Agree
□ Disagree
□ Strongly Disagree
39. Gastric aspirate is an invasive method to use in children
□ Strongly agree
□ Agree
□ Disagree
□ Strongly Disagree
40. I would recommend to stop treatment if a child with TB is feeling better
□ Strongly agree
□ Agree
□ Disagree
□ Strongly disagree
41. Traditional medicine can be used in children treated for TB
□ Strongly agree
□ Agree
□ Disagree
□ Strongly Disagree
42. It is important to conduct contact investigation to identify children with TB or at risk of TB
□ Strongly agree
□ Agree

□ Disagree
□ Strongly disagree
43. Providing TB preventive therapy to children is important
□ Strongly agree
□ Agree
□ Disagree
□ Strongly disagree
Satisfaction with quality of care provided in the health facility
44. The majority of staff in my health facility have adequate training regarding childhood TB
□ Strongly agree
□ Agree
□ Disagree
□ Strongly Disagree
45. Diagnostic tools available in my health facility are adequate for the diagnosis of childhood TB
□ Strongly agree
□ Agree
□ Disagree
□ Strongly Disagree
46. Laboratory services in my health facility or that my health facility uses are adequate for the diagnosis of childhood TB
□ Strongly agree
□ Agree
□ Disagree
□ Strongly Disagree
47. In my health facility, laboratory test results are available on time to be able to decide to treat children for tuberculosis
□ Strongly agree
□ Agree
□ Disagree
□ Strongly Disagree
48. In my health facility, child-friendly fixed dose combination formulations are always available
□ Strongly agree
□ Agree
□ Disagree
□ Strongly disagree
49. In this health centre, it is complicated to refer children with presumptive tuberculosis to another health centre
□ Strongly agree
□ Agree
□ Disagree
□ Strongly Disagree
Community perceptions
50. People in my community believe that a child who has persistent cough should be brought to the clinic as soon as possible
□ Strongly agree
□ Agree
□ Disagree
□ Strongly Disagree
51. People in my community are worried about being in contact with children with TB or presumptive TB
□ Strongly agree
□ Agree
□ Disagree
□ Strongly Disagree
52. People in my community are aware of the availability of tuberculosis services in this health centre
□ Strongly agree

□ Agree
□ Disagree
□ Strongly Disagree

D. Practices

53. How often do you diagnose children with TB or presumptive TB in your health facility?
□ Few times a week
□ Few times a month
□ Few times a year
□ Never
54. To collect sputum for TB diagnosis in a child:
a) You give a container to the mother and you tell her to bring it back with sputum
□ Always
□ Often
□ Sometimes
□ Never
b) You try to collect sputum on the spot/immediately
□ Always
□ Often
□ Sometimes
□ Never
c) You refer to another center for sputum collection
□ Always
□ Often
□ Sometimes
□ Never
d) You don't collect sputum from children
□ Always
□ Often
□ Sometimes
□ Never
55. When a child has presumptive TB and is not able to expectorate sputum:
a) You perform gastric aspirate
□ Always
□ Often
□ Sometimes
□ Never
b) You perform nasopharyngeal aspirate
□ Always
□ Often
□ Sometimes
□ Never
c) You induce expectoration by nebulized hypertonic saline solution
□ Always
□ Often
□ Sometimes
□ Never
d) You collect stool sample
□ Always
□ Often
□ Sometimes
□ Never
e) You don't collect sputum
□ Always
□ Often

□ Sometimes
□ Never
f) You refer the child to higher level health facility
□ Always
□ Often
□ Sometimes
□ Never
57. Do you use the following methods to perform a specimen collection procedure in a child?
a) Restrain the child during the procedure
□ Always
□ Often
□ Sometimes
□ Never
b) Take time to comfort and explain the procedure to the child
□ Always
□ Often
□ Sometimes
□ Never
c) Involve parents during the procedure
□ Always
□ Often
□ Sometimes
□ Never
58. Do you wear personal protective equipment before contact with children with TB or presumptive TB?
□ Always
□ Often
□ Sometimes
□ Never
62 c) You refer the child to facility with paediatrician/specialist
□ Always
□ Often
□ Sometimes
□ Never
63. Do you give advice/education to parents/guardians on what to do when the child throws up the medicine?
□ Always
□ Often
□ Sometimes
□ Never
64. When an adult patient has TB, do you recommend to avoid contact in the household with young children?
□ Always
□ Often
□ Sometimes
□ Never
65. Do you recommend to children with TB or presumptive TB to wear a mask when waiting at the health facility?
□ Always
□ Often
□ Sometimes
□ Never
66. Do you organize or participate in TB awareness or education sessions in your health facility?
□ Always
□ Often
□ Sometimes

□ Never
67. Do you do investigation of contacts of a child newly diagnosed with TB?
□ Always
□ Often
□ Sometimes
□ Never
68. Do you do child contact tracing for newly diagnosed adult TB cases (index case)?
□ Always
□ Often
□ Sometimes
□ Never
A child aged 3 years comes in with a persistent cough of 16 days duration, the cough is dry and the child is not short of breath. According to his mother, the child is less playful, looks very tired and has been feeling the same way for the past four weeks.
70. What likely diagnosis do you suspect?
□ HIV
□ Acute respiratory infection/pneumonia
□ Asthma
□ Tuberculosis

Do you have any problem in your practice?

Questionnaire about TB knowledge for families living in rural villages

<u>Study population:</u> <u>sample of families living in rural villages</u>

- Characteristics of caregivers:
  - demographic (age)
  - demographic (sex)
  - relationship with the child (mother/father/ sister-brother/aunt-uncle/grandparents)
  - relationship with the child (living in the same house/NOT living in the same house)
  - socio-economic status (education): no formal education, primary school, secondary school,advanced school
  - socio-economic status (type of work): agriculture/manufacturing/mining/fishing/energy/craftworker/trade /tourism/teacher /other
  - how many people in the household (number)
  - type of village: rural/urban
  - how many children to take care (number)
  - which age (each child)
- Knowledge of TB disease:
  - Have you ever heard of TB?
  - Do you know anyone in your family and/or in your village who had this disease?
  - Do you know which symptoms are suspected for TB?
  - Do you know that persistent cough > 2 weeks is suspected with TB?
  - Do you know that weigh loss or poor weigh gain is suspected with TB?
  - Do you know that TB is a contagious disease if not treated?
  - Do you know that people living with HIV have an increased risk of TB?
  - Do you know that children have an increased risk of TB ?
  - Do you know that in your country a vaccine is given at birth to any child to prevent severe form of TB?
  - Do you know that TB and HIV care is free?
  - Do you perceive any stigma for having TB?

PART 2 <u>Just for caregiver (>18 years old) of a child diagnosed with TB in his life</u>

- Tuberculosis type:
  - pulmonary /extrapulmonary
- Tuberculosis diagnosis:
  - Why was TB suspected in your child? (symptomatic , TB contact screening, TB screening in HIV positive patient)
  - After how long did you seek care for suspected TB? (weeks)
  - Where did you seek care for suspected TB? (primary health center, district hospital, other)
  - Did your child receive any test to confirm TB diagnosis? Yes /No
  - Test to confirm diagnosis (chest x-ray / blood test / TST / smear test)
- Tuberculosis treatment:

- How long was the child treated for TB? (months)
- With how many drugs? (number)
- Did your child receive any other treatment during the same period? Yes/no
- If yes, what was the treatment? (traditional medicine/ART/non antitubercular antibiotics/vitamins/corticosteroids/bronchodilators /other)
- Did you encounter any challenge in receiving TB treatment for your child? Yes/no
- How was the treatment compliance of your child? (good/poor)
- In case of difficult compliance specify why (refuse of the child/forget medication/medicine not available/ child not at home/medicine not tolerated)
- quantify doses lost of TB treatment (once a week, 2-3 times a week, one week, more than one week)
- How long was the treatment? (weeks)
- Was the treatment stopped early? Yes/no
- Why? (refuse of the child/forget medication/medicine not available/ child not at home/medicine not tolerated)
- Did your child have any side effects of TB treatment ? Yes/no

- Caregivers' perspectives on TB/HIV:
    - Do you know that TB is a contagious disease if not treated?
    - Did you perceive any stigma for having TB?
- HIV status of TB case:
    - HIV test result (positive /negative)
    - HIV test offered as part of TB care (yes / no; only for the patient / for all the family)
    - If HIV positive: did you perceive any stigma for having HIV? Yes/no
    - Did you disclose this condition to your family? Yes/no
    - Did you disclose this condition to other people of your village? Yes/no
    - Did you know that TB and HIV care is free?
